# Effects of A Personalized Intervention Program on the Biochemical and Hematological Profile in Community Dwelling Old Adults—The AGA@4life Intervention Model

**DOI:** 10.3390/ijerph17030718

**Published:** 2020-01-22

**Authors:** Armando Caseiro, Clara Rocha, Ana Margarida Silva, Carla Ferreira, Isabel Silva, Mariana Clemente, Inês Cipriano, Marina Saraiva, Rogério Barreira, Joana Azenha, Maria Helena Loureiro, Anabela Martins, Telmo Pereira

**Affiliations:** 1 Politécnico de Coimbra, ESTESC, Ciências Biomédicas Laboratoriais, Rua 5 de Outubro, 3046-854 Coimbra, Portugal; ana.margarida.silva22@gmail.com (A.M.S.); carlaasferreira_24@hotmail.com (C.F.); isabelnatercia@hotmail.com (I.S.); marianaclemente85@gmail.com (M.C.); rogerf.barreira@gmail.com (R.B.); 2 LABINSAÚDE—Laboratório de Investigação em Ciências Aplicadas à Saúde, Instituto Politécnico de Coimbra, ESTeSC, Rua 5 de Outubro, 3046-854 Coimbra, Portugal; helenasoaresl@gmail.com (M.H.L.); anabelacmartins@estescoimbra.pt (A.M.); telmo@estescoimbra.pt (T.P.); 3 Unidade I&D Química-Física Molecular, Faculdade de Ciências e Tecnologia, Universidade de Coimbra, 3004-535 Coimbra, Portugal; 4 Politécnico de Coimbra, ESTESC, Ciências Complementares, Rua 5 de Outubro, 3046-854 Coimbra, Portugal; clarapr@estescoimbra.pt; 5 INESC Coimbra, Department of Electrical and Computer Engineering, University of Coimbra, Polo 2, 3030-290 Coimbra, Portugal; 6 Politécnico de Coimbra, ESTESC, Fisiologia Clínica, Rua 5 de Outubro, 3046-854 Coimbra, Portugal; inesnoronhacipriano@gmail.com; 7 Politécnico de Coimbra, ESTESC, Fisioterapia, Rua 5 de Outubro, 3046-854 Coimbra, Portugal; marina.saraiva@outlook.com; 8 Serviço de Sangue e Medicina Transfusional, Centro Hospitalar e Universitário de Coimbra, 3004-561 Coimbra, Portugal; 9 Politécnico de Coimbra, ESTESC, Dietética e Nutrição, Rua 5 de Outubro, 3046-854 Coimbra, Portugal; joana.azenha2@gmail.com

**Keywords:** healthy aging, exercise, prevention, cholesterol, hemoglobin

## Abstract

Aging is a social and economic challenge of the highest importance and a multidisciplinary intervention seems to be a promising approach for improving the quality of life of elderly individuals. This project was designed aimed at promoting an active and healthy aging through the implementation of an intervention program based on the comprehensive geriatric assessment model (AGA@4life), focused on promoting health and wellbeing, independence and autonomy, mobility, and social inclusion. A non-randomized interventional study was designed to evaluate the effect of only a dietetic and nutritional approach (control group (CG)) and the combination of a tailored exercise program and a dietetic and nutritional approach (intervention group (IG)) in the biochemical and hematological profile of older adults in the framework of AGA@4life. The 34 participants enrolled, aged 65 years or over, were subject to a thorough baseline (T0) multidisciplinary diagnostic evaluation, including the gathering of clinical information and a battery of biochemical and hematological determinations, and reevaluated after eight weeks of intervention (T1). Between T0 and T1, an increase in albumin and total proteins serum levels were observed in both groups (*p* < 0.01); the hematological profile in CG and IG showed an increase in red cell count and hemoglobin (*p* < 0.05). In IG, an increase of HDL cholesterol (*p* < 0.001) and a decrease of triglycerides (*p* = 0.001) were still observed. The AGA@4life multidisciplinary intervention improved the hematological and biochemical profile of old adults, potentially contributing to delay the development of several aging comorbidities and increase the quality of life of participants.

## 1. Introduction

Aging is a physiological process inherent to living beings, influenced by the interaction of multiple endogenous and exogenous factors that characterize the adaptive biological response and the genetic component [1]. Average life expectancy increased by 5.5 years between 2000 and 2016, with life expectancy at birth around 80.9 years in the constituent countries of the European Union and 78.9 years in the United States of America [2]. Aging is a social and economic challenge of the highest importance; increased life expectancy and declining birth rates are enhancing factors for this demographic transformation process, producing new challenges concerning the elderly’s inclusion in society, their active life expectancy, their quality of life and the sustainability of health, and social security [3]. 

Age is a predictive factor in the development of chronic diseases, such as cardiovascular diseases, cancer, stroke, chronic obstructive pulmonary disease, chronic kidney disease, type 2 diabetes, and Alzheimer’s, responsible for an increased morbidity, hospitalizations, health costs, and mortality [1,2,4,5,6]. Age is also the main risk factor for geriatric syndromes, including fragility and immobility, as well as decreased physical resilience [7]. Advanced age contributes to the exacerbation of chronic systemic inflammation, oxidative stress, DNA damage, decline of mitochondrial function and cellular senescence, thus meeting all the necessary conditions for the onset of metabolic disorders [2,6,7].

Fat tissue plays an important role in mechanisms and pathways involved in longevity and genesis of age-related diseases, including insulin resistance, cardiovascular disease, type 2 diabetes, and osteoporosis. Throughout life, there are major changes in fat distribution and function that affect the body’s metabolism [8,9,10,11]. In addition, advancing age results in the accumulation of adipocytes in the bone marrow cavities also, resulting in loss of hematopoietic activity and disorders of bone loss. Bone marrow mesenchymal stem cells are responsible for the origin of adipocytes and osteoblasts, but with aging they tend to differentiate mostly into adipocytes. Several studies suggest that this process results in impairment of osteogenic and hematopoietic activities [8,12].

At the biochemical level, with regard to renal function, there is a gradual loss of nephrons, decreased enzymatic and metabolic activity of tubular cells, and a higher incidence of pathological processes. In addition to this, there is a decrease in drug excretion, the incidence of renal diseases is higher, and diseases such as hypertension and diabetes also affect renal function, albeit indirectly. In terms of liver function, there is atrophy and a decrease in liver size, as well as decreased function, such as protein synthesis. The ability to eliminate substances is also compromised, which can lead to drug accumulation [13].

Cardiovascular disease remains one of the most important causes of death. The most common cause, atherosclerosis, caused by fat deposition in the vascular walls, has progressively developed over the years. To assess cardiovascular risk, the lipid profile is generally determined [13]. Cholesterol and triglycerides are involved in the process of atherosclerosis and may in the long run lead to more serious lesions (thrombosis, ischemia, and infarction) [14]. Hypercholesterolemia is quite common in the elderly, and there is a strong relationship between high cholesterol levels and the onset of cardiovascular disease [15]. There are several risk factors for increased cholesterol levels, including; a high cholesterol and saturated fat diet, which increases low density lipoproteins (LDL) cholesterol and triglycerides; common intestinal disorders in the elderly, such as decreased intestinal transit which may lead to increased LDL concentration; physical inactivity; frequent metabolic disorders, related to high body fat content and insulin resistance that are associated with lipid alterations; metabolic syndrome; and diabetes mellitus [14,15].

Aging also leads to changes in the primary lymphoid organs, in which there is infiltration of fat in the marrow and atrophy of the thymus, which leads to a reduction in the space allocated to hematopoietic cells [16]. 

Thus, as aging is associated with the development and appearance of various pathologies, the need for a multidisciplinary intervention seems increasingly to be a promising approach for improving the quality of life of elderly individuals. To match this cross-cutting issue to the society, a project was designed aimed at promoting an active and healthy aging through the implementation of an intervention program based on the comprehensive geriatric assessment model (in Portuguese, Abordagem Geriátrica Ampla—AGA@4life) [17,18,19,20]. The AGA@4life model is currently one of the cornerstones in the geriatric approach, being fundamental as a response to the complexity and diversity of problems presented by the elderly [21].

The AGA@4life program is an intervention program based on an individual, holistic, and multidisciplinary assessment protocol, from which intervention strategies will be implemented, adjusted to each participant needs, and aimed at preventing frailty and functional, cognitive, and social decline of the elderly, supported by differentiated and multidisciplinary technical skills. The strategic action of AGA@4life is focused on the valorization of the elderly person, by promoting health and wellbeing, independence and autonomy, mobility, and social inclusion [22,23].

Combining nutritional education, cognitive stimulation, co-morbidities monitoring, physiotherapeutic interventions, therapeutic counseling among others, allows a more diversified and effective action in reducing the aggravation of comorbidities in the elderly. Improvements associated with this type of intervention, even if not very significant due to the metabolic capacity of the elderly, can have a significantly positive impact on improving quality of life and, consequently, reduce mortality [22,23].

This study aimed to evaluate the impact of the AGA@4life program comprehending a nutritional intervention, modifying and correcting dietary errors, while implementing a balanced diet plan adjusted to the nutritional needs of each user, as well as evaluate the impact of a tailored exercise program in biochemical and hematological parameters in old adults in a day care center. 

## 2. Material and Methods

### 2.1. Design and Population

The study involved a multidisciplinary team including a physiotherapist, dietitian, physiologist, psychologists, audiologists, and biomedical laboratory scientists in order to implement the AGA@4life program.

The AGA@4life program comprised six key steps: (1) Data gathering—diagnostic evaluation of the target population; (2) discussion of cases among the multidisciplinary working group; (3) definition of an individualized, multidisciplinary intervention plan; (4) implementation of the intervention plan, in articulation with the elderly, family members, and caregivers; (5) monitoring the response to the intervention plan; (6) revising the intervention according to the results. 

This work consisted in a non-randomized interventional study designed to evaluate the effect of a tailored exercise program and a dietetic and nutritional approach in the biochemical and hematological profile of older adults in the framework of AGA@4life project. The 34 participants enrolled were recruited from a day care centre in Coimbra district, Portugal, and were aged 65 years or over, were of female and male genders, were physically autonomous, and had no prior history of cerebrovascular or neurological disorders.

A baseline multidisciplinary diagnostic evaluation (T0) of each participant was performed, including the gathering of clinical and demographic information, namely data on comorbidities, diet and physical activity profile, cardiovascular risk profile, ongoing treatments, functional ability, and a battery of biochemical and hematological determinations including: albumin, total proteins, total bilirubin, total cholesterol, high density lipoproteins (HDL) cholesterol, LDL cholesterol, glucose, creatinine, uric acid, urea, triglycerides, alanine aminotransferase, aspartate aminotransferase, amylase, lactate dehydrogenase, alkaline phosphatase, GGT, creatine kinase, calcium, C-reactive protein, and a complete blood count.

For the determination of blood parameters, we collected 10 ml of peripheral blood by venous puncture for serum and plasma EDTA tubes. The biochemical profile was determined by spectrophotometry in the chemistry analyzer Prestige 24i (Tokyo Boeky, Tokyo, Japan) with reagents from Cormay^®^ (Warsaw, Poland) The total blood count were performed in a hematology analyzer Cell-Dyn Sapphire™ (Abbott Laboratories, Santa Clara, CA, USA). All the assays were validated and passed the quality control.

The nutritional intervention included the application of different tools at baseline evaluation and after the program implementation, namely the Mini Nutritional Assessment (MNA), validated in Portugal, to determine nutritional risk; Food Frequency Questionnaire, validated in Portugal, to determine intake history and food frequency; 24-h survey to determine food intake of the last 24 hours. The anthropometric measures: Height, weight, body mass index (BMI), waist circumference, arm circumference, hip waist ratio, and leg circumference; were determined using a non-extensible tape measure. Body composition, specifically the fat mass percentage and lean mass percentage were determined using bioelectrical impedance analysis (BIA) [24,25].

The nutritional intervention consisted in the first phase in the evaluation of the nutritional risk of each participant by MNA and anthropometric data collection: BMI, perimeter and lean and fat mass; clinical history; food anamnesis. The collected information enabled the calculation of the nutritional needs of each user and the elaboration of an individualized dietary plan, taking into account the pathologies associated with the clinical history of the user and the anamnesis and biochemical parameters, as well as the elaboration of a set of recommendations to correct the main dietary errors observed and possible risk factors, such as hypertension, type 2 diabetes mellitus, among others. For each day-care user, the appropriate eating plan was delivered and explained individually. The implementation of a nutritional intervention also included an audit to the production of meals and, according to these results, training was given to those responsible for preparing day center meals. This training covered topics such as healthy eating, specific nutritional needs for the elderly, awareness of salt reduction, and substitution with aromatic herbs [24,25].

At the level of the established meals in the day care center, they were evaluated taking into account the proposal of a qualitative assessment tool for menus aimed at the elderly, published in March 2017 by the National Program for the Promotion of Healthy Eating. Taking into account these guidelines, a new menu plan was drawn up and instituted. A diet manual was developed including a set of different types of diets, programmed and typified, with an indication of prohibited and allowed foods and confections, that result from the nutritional needs of the different users assisted in the center.

The participants were divided in two groups according to their willingness to integrate the exercise program: The control group (CG) without exercise program intervention (*n* = 16); and the intervention group (IG) with outdoor aerobic exercise sessions and an exercise program based on the OTAGO and adapted to a technological system with Exergames, in the functioning of older adults living in the community (*n* = 18). 

After the baseline characterization (T0), the CG was encouraged to keep the usual daily routines, while the IG was submitted to a tailored exercise plan based on aerobic exercise sessions and on the OTAGO incorporated in a technological system (FallSensing) with pressure and inertial sensors, feedback, and Exergames for 8 weeks, three times a week, lasting approximately 20 min. This program targeted the knee flexors and extensors muscles, hip abductors, ankle dorsi flexor/plantar flexor muscles, and balance. The aerobic exercise sessions followed the recommendations of the World Health Organization [26] consisting of two sessions per week with the following structure: A 10-min walk on flat or slightly sloping ground, balance and coordination exercises, and joint mobilization exercises with respiratory coordination (total session duration of 1 h).

After the eight weeks (T1), all the participants that completed the program (*n* = 18; 12 females, 6 males; average age 80.2 ± 10.6) were re-assessed, repeating the evaluations performed at the baseline evaluation. At the end of the program, the CG was composed of 7 participants and the IG of 11 participants. During this period, we had some drop outs, namely justified by hospital internments and exchange of day center.

### 2.2. Statistics

Statistical analysis was performed using software IBM SPSS^®^ v.24 (National Opinion Research Center, Chicago, IL, USA) and GraphPad Prism v. 6.04 (La Jolla, San Diego, CA, USA).

All continuous results are expressed as a mean ± standard deviation (SD), and to evaluate the normality of distribution, the Shapiro–Wilk test was used. The student’s t-test was applied for baseline group comparisons when distribution was normal, otherwise the Wilcoxon test was used. Differences were considered statistically significant at *p* value less than 0.05.

### 2.3. Ethics

The study was conducted according to the guidelines of the Declaration of Helsinki and approved by the Ethics Committee of the Polytechnic Institute of Coimbra (approval 8/2018). The anonymity and confidentiality of the collected data were guaranteed and all participants signed an informed consent prior to the study. There is no conflict of interest to be declared.

## 3. Results

The participants that completed the AGA@4life program (*n* = 18; 12 females, 6 males; average age 80.2 ± 10.6) from the two groups; the CG (*n* = 7) and IG (*n* = 11); were evaluated in the different moments (T0 and T1) for biochemical and hematological parameters, and the differences between the two moments and groups were analyzed by statistical tests.

**Figure 1:**
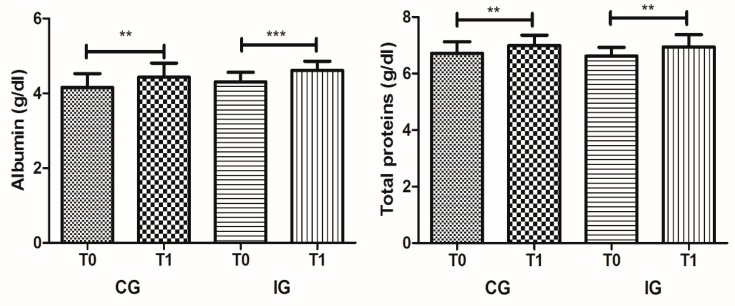
Albumin and total protein serum levels in the control group (CG) and intervention group (IG) at T0 and T1 moments (** *p* < 0.01; *** *p* < 0.001).

The impact of dietetic and nutritional intervention on biochemical parameters is described in Table 1. The data show the evolution of the different biochemical parameters in CG between T0 and T1, demonstrating significant differences; namely, an increase in albumin, with 4.16 ± 0.37 g/dL in T0 and 4.43 ± 0.37 in T1 (*p* = 0.002), and in total proteins, with 6.71 ± 0.41 in T0 and 6.99 ± 0.36 (*p* = 0.003) (Figure 1). The calcium levels also showed a slight reduction from 8.80 ± 0.36 to 8.49 ± 0.41 mg/dL, however, remained within the recommended reference values (8–11 mg/dL). The evaluation of enzyme activities, renal function, and lipid profiles did not present statistically significant alterations.

The hematological profile (Table 2) also reflected the dietetic and nutrition intervention in CG and we observed a statistically significant increase in red cell blood count from 4.40 ± 0.38 to 4.70 ± 0.47 × 10^12^/L (*p* = 0.007) and hemoglobin from 127.3 ± 20.1 to 134.4 ± 22.8 g/L (*p* = 0.04) between T0 and T1, respectively. In CG, we also observed a slight reduction in neutrophils count, from 5.61 ± 1.84 to 5.35 ± 1.99 (*p* = 0.016).

**Figure 2:**
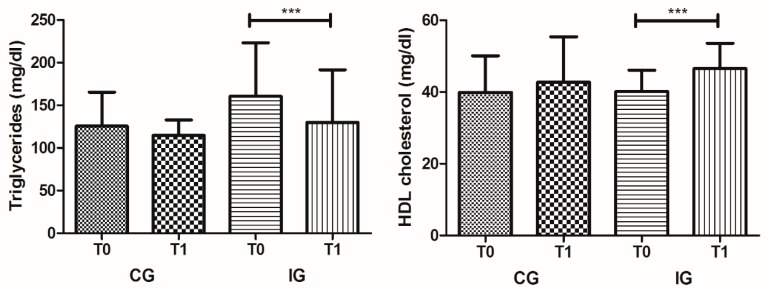
HDL cholesterol and triglycerides serum levels in CG and IG at T0 and T1 moments (*** *p* < 0.001).

With respect to IG, we observed the joint impact of dietetic and nutritional intervention and exercise on biochemical parameters (Table 1). This intervention showed a greater impact on the modification of biochemical parameters, namely in lipid profile, with a significant increase of HDL cholesterol from 40.11 ± 5.98 at T0 to 46.54 ± 7.03 in T1 (*p* < 0.001) and a decrease of triglycerides from 160.49 ± 62.78 at T0 to 129.99 ± 61.62 in T1 (*p* = 0.001) (Figure 2). The beneficial impact on albumin and total protein serum levels was also verified in IG, like in CG, with a significant increase among T0 to T1, from 4.31 ± 0.25 to 4.61 ± 0.25 mg/dL (*p* < 0.001) and 6.62 ± 0.31 to 6.93 ± 0.44 mg/dL (*p* = 0.004), respectively (Figure 1). The uric acid serum levels also showed a small reduction between T0 and T1 (*p* = 0.022), from 5.47 ± 1.76 to 4.94 ± 1.58 mg/dL, respectively.

In the IG, the combination of dietetic and nutritional intervention and exercise produced similar results to CG in relation to hematological profiles (Table 2), with a statistically significant increase being observed, between T0 and T1, in red cell blood count from 3.98 ± 0.48 to 4.29 ± 0.54 × 10^12^/L (*p* = 0.013), hemoglobin from 127.0 ± 18.1 to 135.6 ± 17.4 g/L (*p* = 0.031), and hematocrit from 0.3797 ± 0.0533 to 0.4096 ± 0.06.0 L/L (*p* = 0.025).

## 4. Discussion

The biological, psychological, and social context of the elderly is very specific. Therefore, the physiological processes associated with the course of aging are key determinants of the effectiveness of any program aimed at promoting an active and healthy person [28,29]. This population is of a remarkable complexity, with multi-morbidity as common feature and the coexistence of several chronic diseases associated with the insidious loss of functionality and the increased risk of mortality [30,31]. Considering the particular features of this target population, it seems clear that any plan or action to confront it must rely on an individualized, multidimensional, and interdisciplinary approach, which are the basis of AGA@4life program.

There are several laboratory tests that allow us to evaluate the physiological functions of the individuals, enabling us to understand the state of renal, hepatic, cardiac, and hematopoietic function. In this work, we evaluated the impact of AGA@4life program on biochemical and hematological profiles of the participants [32,33].

The impact of dietetic and nutritional interventions on biochemical parameters were observed, between the evaluation moments (T0 and T1), with significant increases in the albumin and total protein serum levels of the CG participants. These results demonstrate the contribution of adjusted institutional feeding that reflects the basic principles of healthy eating, based on the guidelines of Mediterranean Food Wheel provided to all individuals. A protein-deficient diet with adjusted calories may lead to an important decrease of blood albumin levels, with even more noticeable reflexes in body weight reduction. Hepatic albumin synthesis is affected by protein–calorie malnutrition; this adjusted nutrient intake is relevant for polysomal aggregation and maintenance of cellular RNA levels needed for albumin synthesis [34]. The evaluation on enzyme activities, renal function, and lipid profiles did not present statistically significant alterations, indicating that the intervention did not cause any disadvantages for the participants.

With respect to IG, the joint impact of dietetic and nutritional intervention and exercise on biochemical parameters showed a greater impact on the modification of lipid profiles, with an increase of HDL cholesterol and a remarkable decrease of triglycerides. The c-HDL is a lipoprotein capable of transporting cholesterol from peripheral areas to the liver, thus having a protective function against CVD and atherosclerosis [35,36]. Increased levels of serum triglycerides are associated with a higher percentage of visceral adipose tissue and consequent comorbidities [37,38,39] Atherosclerosis, obesity, and diabetes mellitus type 2 are characterized by high concentrations of triglycerides, LDL cholesterol and low HDL cholesterol [37,39,40]. Several studies have increasingly described the relevance of regular exercise in health, in the prevention of comorbidities, and even in reducing mortality. Exercise benefits include improvements in cognitive and mental health, loss of body fat mass, increased lean body mass, and improvements in diabetes and hypertension, among others [41,42,43,44].

The beneficial impact of the intervention on albumin and total protein serum levels was also verified in IG and also verified by a reduction in uric acid serum levels. Several studies have observed that there is a tendency for uric acid to increase, with a higher incidence in males, and that urea may be slightly increased in the elderly due to the inability of the kidneys to excrete it [32,45,46,47]. The AGA@4life program enabled the reduction of uric acid serum levels, however, urea levels were slightly elevated as expected in the participants, and its levels did not change with the intervention.

With respect to liver transaminases, they apparently do not show significant changes with aging, and there may only be a slight increase in aspartate aminotransferase [48]. Our study showed that both evaluated groups (CG and IG) presented values within reference ranges. However, another study states that alanine aminotransferase tend to decrease with age and can be considered a marker of aging [26]. Concerning bilirubin serum levels, they are thought to increase slightly due to the inability of liver function excrete them [49]. Our participants also presented total bilirubin serum levels within the reference range. 

The AGA@4life program also presented significant modifications in hematological parameters evaluated. In CG, individuals were found to have a statistically significant improvement in some hematological parameters like red cell blood count and hemoglobin. In CG, we also observed a slight reduction in neutrophils count. In the other hematological parameters, we did not observe significant changes in CG participants. White blood cell count tends to be normal, however, in the most fragile elderly, an increase in neutrophil count and a slight decrease in lymphocyte count may be observed. Regarding platelet levels, no significant changes are described [16].

In the IG, the association of dietetic and nutritional intervention and exercise produce similar results to CG in relation to hematological profiles, being observed a significantly increase, in red cell blood counts, hemoglobin, and even more in hematocrit, emphasizing the potential role of exercise in this study group.

Aging leads to changes in the primary lymphoid organs, in which there is infiltration of fat in the marrow and atrophy of the thymus, which leads to a reduction in the space allocated to hematopoietic cells [16]. Hemoglobin values tend to decrease due to reduced oxygen demand as a result of decreased body mass, physical activity, among a number of other factors [50]. With this, anemia is present in a small percentage of individuals over 65 years old; but in the elderly living in households, the prevalence is much higher. It is usually a mild to moderate normocytic anemia with a low reticulocyte index [50,51]. Although not translating to a severe clinical condition, these changes affect the quality of life of the geriatric population, being related to the increase in the number of falls, depression, slower walking speed, loss of mobility, functional impairment, reduced grip strength, worsening of comorbidities, and mortality [16].

The authors consider findings to be relevant at the hematological and biochemical levels for the improvement of the quality of life in ageing people, however, they emphasize the need to extend the intervention program to more day care centers, increase the number of participants, and evaluate other geographic locations in order to consolidate the results.

## 5. Conclusions

The AGA@4life program, a multidisciplinary intervention study in older adults, enables the improvement of hematological and biochemical profiles of the participants. The joint impact of dietetic and nutritional intervention and exercise on biochemical parameters promoted the modification of lipid profiles, with an increase in HDL cholesterol and a remarkable decrease in triglycerides. At the hematological level, the program enabled the increase of red cell blood counts, hemoglobin, and hematocrit values. These improvements at the physiological level potentially contribute to delaying the development of several aging comorbidities and increase the quality of life of the older people, with a cost reduction for health-care services.

## Figures and Tables

**Table 1:** Biochemical parameters measured in CG and IG at moments T0 and T1, presented in medium values ± standard deviation and respective reference range values.

Parameters	Units	CG (*n* = 7)			IG (*n* = 11)			Reference Values	
		T0	T1	*p*	T0	T1	*p*	Male	Female
Albumin	g/dL	4.16 ± 0.37	4.43 ± 0.37	0.002	4.31 ± 0.25	4.61 ± 0.25	<0.001	3.0–5.0	3.0–5.0
Total proteins	g/dL	6.71 ± 0.41	6.99 ± 0.36	0.003	6.62 ± 0.31	6.93 ± 0.44	0.004	6.0–8.0	6.0–8.0
Total bilirubin	g/dL	0.51 ± 0.15	0.54 ± 0.14	n.s	0.60 ± 0.30	0.56 ± 0.18	n.s	0.3–1.2	0.3–1.2
Total cholesterol	mg/dL	178.90 ± 55.62	177.89 ± 33.97	n.s	180.23 ± 35.18	181.69 ± 33.98	n.s	<200	<200
HDL cholesterol	mg/dL	39.86 ± 10.21	42.70 ± 12.67	n.s	40.11 ± 5.98	46.54 ± 7.03	<0.001	>55	>60
LDL cholesterol	mg/dL	130.69 ± 48.87	125.22 ± 27.14	n.s	128.95 ± 33.65	123.83 ± 33.33	n.s	<100	<100
Glucose	mg/dL	107.81 ± 39.85	106.37 ± 29.35	n.s	126.91 ± 91.63	121.77 ± 61.56	n.s	74–106	74–106
Creatinine	mg/dL	0.72 ± 0.12	0.77 ± 0.21	n.s	0.77 ± 0.16	0.73 ± 0.12	n.s	0.7–1.3	0.6–1.1
Uric acid	mg/dL	5.79 ± 1.31	6.00 ± 1.48	n.s	5.47 ± 1.76	4.94 ± 1.58	0.022	3.6–7.7	2.5–6.8
Urea	mg/dL	41.77 ± 16.10	49.84 ± 24.59	n.s	55.84 ± 14.30	56.64 ± 12.45	n.s	<50	<50
Triglycerides	mg/dL	125.60 ± 39.75	114.77 ± 18.12	n.s	160.49 ± 62.78	129.99 ± 61.62	0.001	<200	<200
Alanine aminotransferase	IU/L	23.06 ± 15.70	23.19 ± 13.71	n.s	19.33 ± 5.54	19.51 ± 7.32	n.s	<42	<32
Aspartate aminotransferase	IU/L	22.40 ± 12.37	22.43 ± 10.12	n.s	20.78 ± 6.14	19.73 ± 4.93	n.s	<37	<31
Amylase	IU/L	53.59 ± 14.03	58.55 ± 17.74	n.s	48.04 ± 25.75	70.01 ± 35.99	n.s	28–100	28–100
Lactate dehydrogenase	IU/L	377.47 ± 115.74	373.96 ± 120.01	n.s	361.29 ± 64.70	374.38 ± 52.97	n.s	225–450	225–450
Alkaline phosphatase	IU/L	116.71 ± 78.85	105.71 ± 58.63	n.s	65.55 ± 30.03	66.55 ± 21.54	n.s	56–119	53–141
γ-glutamyl transferase	IU/L	34.50 ± 24.72	32.66 ± 19.42	n.s	35.41 ± 35.12	31.43 ± 31.24	n.s	<55	<40
Creatine kinase	IU/L	86.29 ± 47.32	100.97 ± 45.75	n.s	87.62 ± 43.87	69.62 ± 29.47	n.s	<190	<167
Calcium	mg/dL	8.80 ± 0.36	8.49 ± 0.41	0.020	8.53 ± 0.38	8.36 ± 0.40	n.s	8–11	8–11
C-reactive protein	mg/L	1.05 ± 1.05	1.19 ± 2.01	n.s	0.25 ± 0.19	0.49 ± 0.83	n.s	<5	<5

n.s.—non statistically significant (*p* > 0.05).

**Table 2:** Hematological parameters measured in CG and IG at moments T0 and T1, presented in medium values ± standard deviation and respective reference range values.

Parameters	Units	CG (*n* = 7)			IG (*n* = 11)			Reference Values [27]	
		T0	T1	*p*	T0	T1	*p*	Male	Female
WBC	× 10^9^/L	9.17 ± 1.82	9.04 ± 1.84	n.s	5.94 ± 1.01	6.27 ± 1.71	n.s	4–10	4–10
NEU	× 10^9^/L	5.61 ± 1.84	5.35 ± 1.99	0.016	3.58 ± 0.64	4.00 ± 1.29	n.s	2–7	2–7
LYM	× 10^9^/L	2.28 ± 0.84	2.48 ± 1.00	n.s	1.75 ± 0.70	1.63 ± 0.54	n.s	1.0–3.0	1.0–3.0
MON	× 10^9^/L	0.71 ± 0.32	0.67 ± 0.24	n.s	0.43 ± 0.12	0.48 ± 0.17	n.s	0.2–1.0	0.2–1.0
EOS	× 10^9^/L	0.49 ± 0.41	0.49 ± 0.47	n.s	0.13 ± 0.06	0.11 ± 0.06	n.s	0.02–0.5	0.02–0.5
BAS	× 10^9^/L	0.07 ± 0.04	0.06 ± 0.03	n.s	0.05 ± 0.03	0.05 ± 0.03	n.s	0.02–0.1	0.02–0.1
RBCi	× 10^12^/L	4.41 ± 0.41	4.76 ± 0.52	0.005	3.97 ± 0.52	4.31 ± 0.56	0.012	4.5–5.5	3.8–4.8
RBCo	× 10^12^/L	4.40 ± 0.38	4.70 ± 0.47	0.007	3.98 ± 0.48	4.29 ± 0.54	0.013	4.5–5.5	3.8–4.8
HGB	g/L	127.3 ± 20.1	134.4 ± 22.8	0.040	127.0 ± 18.1	135.6 ± 17.4	0.031	130–170	120–150
MCV	fL	89.67 ± 7.70	87.47 ± 8.76	n.s	95.75 ± 4.94	94.99 ± 4.69	n.s	80–100	80–100
RDW	%	12.76 ± 1.44	14.23 ± 3.08	n.s	12.20 ± 0.71	12.24 ± 0.91	n.s	11.6–14	11.6–14
MCH	pg	28.79 ± 3.16	28.23 ± 3.38	n.s	31.98 ± 1.96	31.53 ± 1.68	n.s	27–32	27–32
MCHC	g/L	320,7 ± 10,00	322,3 ± 10.05	n.s	334.5 ± 10.64	331.9 ± 10.38	n.s	320–350	320–350
HCT	L/L	0.3963 ± 0.0548	0.4166 ± 0.0655	n.s	0.3797 ± 0.0533	0.4096 ± 0.06.0	0.025	0.40–0.50	0.36–0.46
PLT	× 10^9^/L	281.50 ± 110.54	274.04 ± 68.23	n.s	202.49 ± 53.01	216.31 ± 47.53	n.s	150–400	150–400
MPV	fL	8.07 ± 0.45	8.92 ± 1.17	n.s	8.26 ± 0.86	8.43 ± 0.95	n.s	8.17–9.65	8.17–9.65
PCT	%	0.22 ± 0.08	0.24 ± 0.06	n.s	0.17 ± 0.04	0.18 ± 0.04	n.s	0.17–0.29	0.17–0.29
PDW	GSD	15.89 ± 0.46	16.23 ± 0.56	n.s	16.23 ± 0.59	16.51 ± 0.60	n.s	14.7–17.4	14.7–17.4

WBC—White Blood Cells; NEU—Neutrophils; LYM—Lymphocytes; MON—Monocytes; EOS—eosinophils; BAS—Basophils; RBCi—Impedance measurement of red blood cells; RBCo—Optical measurement of red blood cells; HGB—Hemoglobin; MCV—Mean corpuscular volume; RDW—Red cell distribution width; MCH—Mean corpuscular hemoglobin; MCHC—Mean corpuscular hemoglobin concentration-; HCT—Hematocrit; PLT—Platelet count; MPV—Mean platelet volume; PCT—Plateletcrit; PDW—Platelet distribution width.
